# Brown tumor complicating end-stage kidney disease 

**DOI:** 10.5414/CNCS110195

**Published:** 2020-10-12

**Authors:** Michael Wiederkehr

**Affiliations:** Department of Internal Medicine, Texas A&M Health Science Center, College of Medicine, Baylor University Medical Center, Dallas, TX, USA

**Keywords:** hyperparathyroidism, brown tumor, spinal cord compression, end-stage kidney disease, mineral and bone disorder, metabolic bone disease, case report

## Abstract

Longstanding, severe hyperparathyroidism (HPT) can lead to the formation of “brown tumors”. A brown tumor is a radiolucent bone lesion that is locally destructive; it is not a neoplasm, but rather a stromal mass consisting of fibrous tissue, poorly mineralized woven bone, and supporting vasculature. These tumors are a rare complication of advanced primary or secondary HPT. We present a young female with chronic kidney disease (CKD) on hemodialysis with uncontrolled secondary HPT (SHPT). The patient presented with progressive lower extremity weakness and back pain. CT imaging showed multiple lytic bone lesions involving several ribs and the spine. Subsequent MRI imaging of the thoracic and lumbar spine confirmed expansile bone lesions consistent with brown tumors. One mass protruded into the spinal canal causing severe stenosis at T3 with underlying cord edema. The other lesion at T12 caused only moderate spinal canal stenosis. Our patient underwent urgent neurosurgical resection of the tumor at T3 followed by subtotal parathyroidectomy (PTX).

## Introduction 

Hyperparathyroidism (HPT) is the result of excessive secretion of parathyroid hormone (PTH) by the parathyroid glands, and three different forms are recognized: Primary, secondary, and tertiary. Primary HPT is typically due to an adenoma of the parathyroid glands. While chronic kidney disease (CKD) and end-stage kidney disease (ESKD) are the most common causes of secondary HPT, it can also result from other conditions, in particular when hypocalcemia is present, such as in various forms of gastrointestinal malabsorption (chronic pancreatitis with steatorrhea, small bowel disease), severe vitamin D deficiency, or rarely, due to inadequate dietary intake of calcium [1]. 

Traditionally, the effect of CKD on bone has been classified into distinct categories in dialysis patients, in particular osteitis fibrosa (high bone turnover), adynamic bone disease (low bone turnover), osteomalacia (inadequate bone mineralization), and mixed forms [2, 3]. 

A 2003 National Kidney Foundation Controversies Conference on Mineral Metabolism and Bone Disease in CKD (CKD-MBD) proposed a new deﬁnition for renal osteodystrophy [3]. A follow-up 2006 consensus conference expanded that definition to include extraskeletal calciﬁcations. The term renal osteodystrophy should be used exclusively to deﬁne the bone pathology associated with CKD. Though now uncommonly performed, bone biopsies remain a powerful diagnostic tool. Using three key histologic descriptors – bone turnover, mineralization, and volume (“TMV”), it better defines the pathologic abnormalities and thus the underlying pathophysiology, and helps guide therapy [4]. 

In most individuals, persistently high PTH levels produce a high-turnover bone disease due to activation of the osteoclasts and proliferation of fibroblasts (osteitis fibrosa) [3, 4]. The formation of a brown tumor can be considered the localized expression of an extreme form of high-turnover bone disease. When they form, patients usually remain asymptomatic,and brown tumors are an incidental finding [5]. Here we describe an unusual case of acute spinal cord compression caused by a brown tumor. 

## Case presentation 

**Figure 1.**
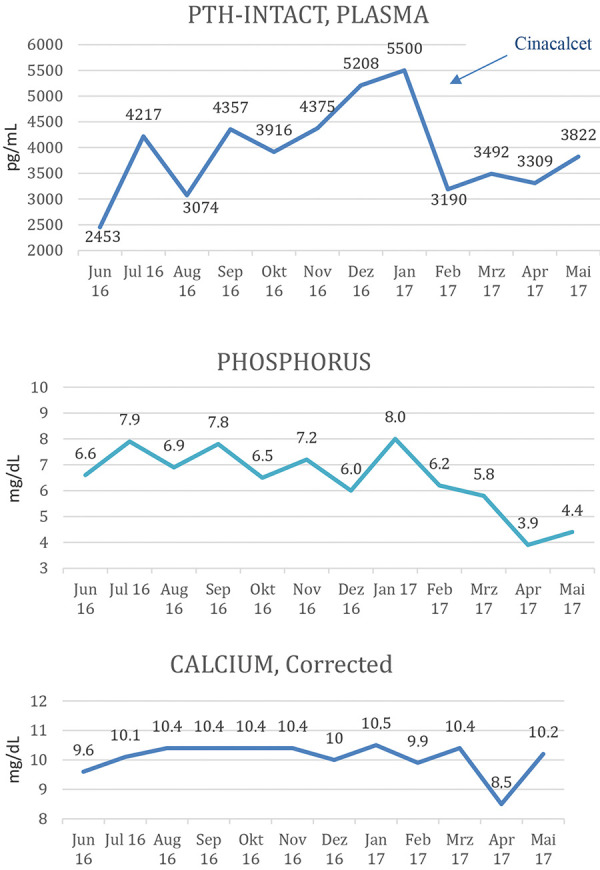
PTH, calcium, and phosphorus levels during the preceding 12 months.

A 33-year-old Hispanic female, dialysis dependent for the past 9 years, presented with acute exacerbation of chronic back and chest pain, bilateral lower extremity weakness, and diffuse skeletal pain. She was anuric and reported no fecal incontinence. Her social situation had been unstable, and adherence with dialysis treatments and medications was poor. HPT developed and became progressively more severe. The data for the 12 months from June 2016 to May 2017 are presented in Figure 1. Intact PTH levels were consistently above 2,000 pg/mL, with a gradual increase to a maximum of 5,500 pg/mL in January 2017. Cinacalcet was initiated at a dose of 120 mg daily. This led to a dramatic drop of intact PTH levels to 3,190 pg/mL in February 2017, though it subsequently increased again to 3,822 pg/mL in May 2017. Likewise, chronic hyperphosphatemia improved from 8.0 to 6.2 mg/dL from January to February 2017 with subsequent normalization (Figure 1). Calcium levels were less consistent. She remained hypercalcemic most of the time (tertiary HPT), though there was a brief drop in serum calcium with initiation of cinacalcet. Interestingly, there was a consistent increase in alkaline phosphate, which continued unabated even after cinacalcet initiation and improvement of PTH. 

**Figure 2.**
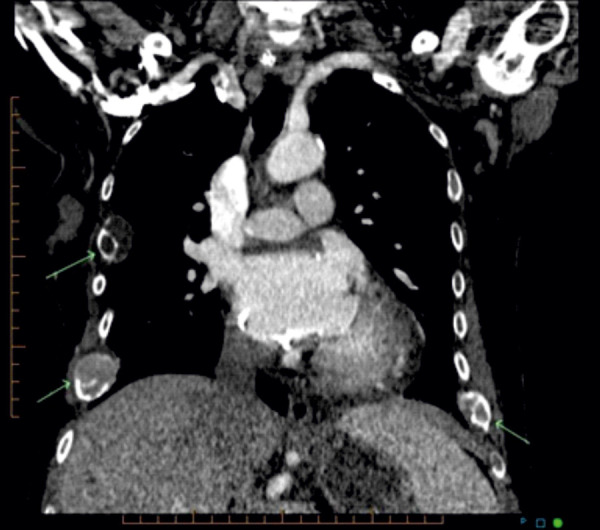
CT of the chest: Lytic bone lesions in ribs with soft tissue masses.

**Figure 3.**
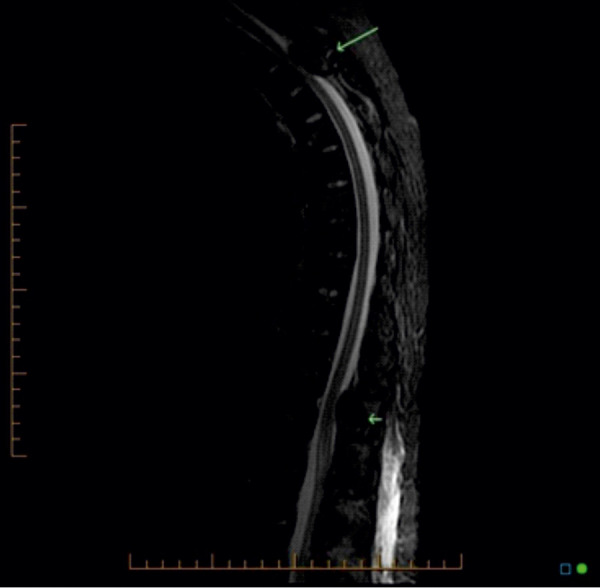
MRI of thoracic spine: Severe spinal canal stenosis and cord edema at T3, moderate stenosis at T12.

On exam, she had no obvious skeletal deformities, but a palpable tender “mass” along the right lower rib cage and localized tenderness over the thoracic, but not the lumbar spine. She was neurologically intact. Straight leg rise was normal. Muscle strength was intact at 5/5 and symmetrical of both upper and lower extremities. Her laboratories were significant for a calcium of 10.3 mg/dL (normal range 8.5 – 10.1 mg/dL), a phosphorus of 4 mg/dL (normal), and an intact PTH of 2,476 pg/mL (normal range 12 – 88 pg/mL). Her alkaline phosphatase was markedly elevated at 519 U/L (normal range 45 – 117 U/L); all her liver function tests were normal. A chest X-ray revealed cardiomegaly and diffuse osteopenia with lucent lesions in the proximal left humerus and right humeral head. Subsequent CT chest with intravenous contrast showed multiple lytic bone lesions involving several ribs and the spine (Figure 2). Two expansile lesions of the spine protruded into the spinal canal causing severe stenosis at T3 and mild narrowing at T12. CT neck soft tissue with and without contrast showed three parathyroid nodules with washout characteristics compatible with parathyroid adenomas, two on the right and one on the left. Imaging also revealed “innumerable” and widespread expansile bone tumors, presumably representing brown tumors, throughout the visualized skeleton. The lesions mainly involved the posterior elements of the cervical and thoracic spine, vertebral bodies, numerous ribs, and the sternum. It again showed the epidural encroachment of tumor at T3 resulting in severe spinal canal stenosis and likely cord compression. Subsequent MRI imaging confirmed expansile masses compatible with brown tumors at T3 with severe spinal canal stenosis and underlying cord edema, and T12 with moderate spinal canal stenosis (Figure 3). 

**Figure 4.**
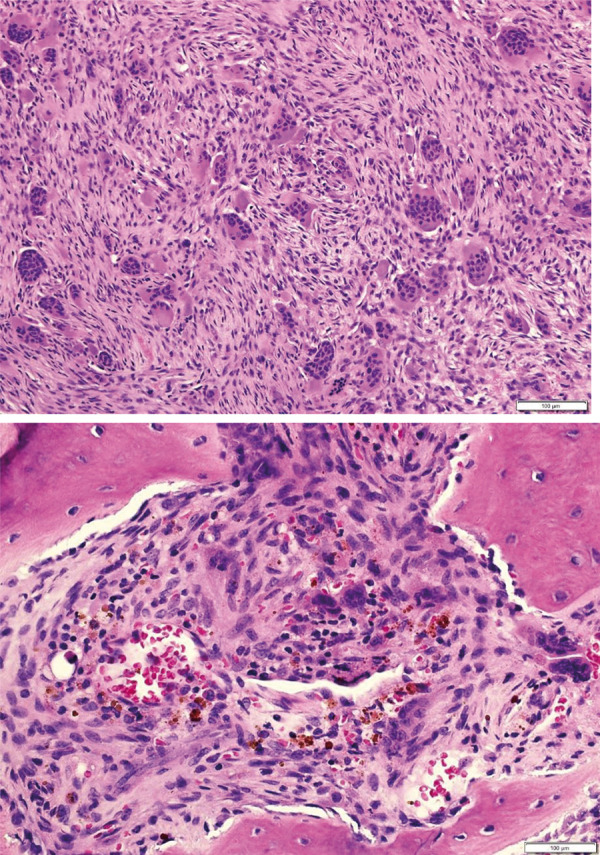
Biopsy of resected bone and stromal mass at T3 consistent with brown tumor.

Despite the absence of focal neurological deficits, our patient had severe pain and bilateral lower extremity weakness. The main concern was the tumor protruding into the spinal canal at T3, causing severe stenosis and spinal cord edema. Therefore, she underwent urgent T2-T4 laminectomy with resection of the tumor at T3. No intervention was made at T12, as there was no cord compression. Intraoperative evaluation of a specimen showed a 4.0 × 2.0 × 0.5 cm aggregate of multiple red soft tissue fragments with a preliminary diagnosis of “spindle cells plus numerous giant cell proliferation”. A tissue specimen submitted in formalin was a 5.5 × 4.0 × 2.0 cm aggregate of tan-brown soft tissue fragments, mixed with elongated stromal cells. The bone showed remodeling with increased osteoid rimming (Figure 4). 

One week later, she underwent subtotal parathyroidectomy (PTX). Three glands were resected, described as “hypercellular parathyroid gland” measuring 2.5 g (right lower), 3.27 g (right upper), and 2.43 g (left upper). The left lower parathyroid gland was much smaller and normal sized at 0.06 g (normal parathyroid glands weigh between 0.02 and 0.16 g) and not resected. Of note, our patient’s three enlarged glands had a combined weight of 8.2 g, almost 70-fold the weight of four regularly sized parathyroid glands. Pre-operative PTH dropped from 2,358 to 268 pg/mL right after surgery, to 11 pg/mL the following day, but improved to 39 pg/mL 8 days postoperatively. We expected a significant decrease in serum calcium due to the sudden drop of PTH as a consequence of a brisk uptake of calcium, phosphorus, and magnesium into her bones (“hungry bones”). To prevent this, we instituted an intravenous continuous infusion of 10% calcium gluconate at 4.6 mEq/h, calcitriol 1.0 µg twice daily, plus vitamin D3 5,000 units daily. Over the next 5 days, we slowly transitioned from intravenous calcium to oral calcium citrate at a dose of 3,800 mg every 6 hours, taken between meals. With these measures, we were able to maintain her serum calcium at or near normal values. For hypophosphatemia, she received a diet unrestricted in phosphorus, and supplemented with milk and potassium/sodium phosphate powder. 

A bone density scan (DEXA) confirmed diffuse bone demineralization with osteoporosis at the lumbar spine (T score –3.54) and osteopenia of the right and left femoral neck (mean T score –1.35). She recovered well from her surgeries but continued to be severely deconditioned and started inpatient rehabilitation. Three weeks later, she returned home, fully functional, neurologically intact, and with a much-improved sense of well-being. 

## Discussion 

The pathogenesis of secondary HPT (SHPT) is complex and remains incompletely understood. It is an intricate interplay of various factors such as hypocalcemia, vitamin D deficiency, reduced formation of active vitamin D (calcitriol), and phosphate retention [2, 3]. 

The increased serum phosphorus stimulates two hormones: PTH from the parathyroid gland, and fibroblast growth factor 23 (FGF23) produced in the bone by osteocytes and osteoblasts. FGF23 lowers serum phosphate by reducing its re-absorption in the renal proximal tubule by down-regulating sodium–phosphate cotransporter (NaPi) [6]. In addition, it reduces serum calcitriol synthesis by inhibition of the renal 1-α-hydroxylase enzyme and promoting its catabolism through stimulation of the 24-hydroxylase enzyme. Despite the elevated levels of FGF23, it is unable to enhance phosphate excretion due to decreasing levels of membrane-bound klotho, a cofactor that augments the affinity of FGF23 to exert its effect on the fibroblast growth factor receptor (FGFR1). Finally, a decrease in calcitriol exacerbates hypocalcemia as intestinal absorption of calcium decreases [7, 8, 9, 10]. 

The parathyroid gland undergoes diffuse polyclonal hyperplasia, often followed by monoclonal nodular hyperplasia. The proliferation of these cells is associated with a decrease in the expression of key regulators of cellular PTH release: the calcium-sensing receptor (CaSR), the vitamin D receptor (VDR), and FGFR1. Thus, the nodular transformation in advanced SHPT decreases the inhibitory effect of calcium and exogenous administration of active vitamin D on PTH [7]. FGF23 per se inhibits PTH secretion and its levels increase throughout CKD, becoming very high in ESKD. However, the reduction in FGFR1 and low levels of klotho preclude adequate cellular inhibition of PTH release [11]. The combination of these factors contribute to a relentless rise in parathyroid levels. 

Brown tumors in ESKD develop in persons with very high PTH levels, usually in excess of 10 times the upper limit of normal, and over a prolonged period of time [12, 13]. A brown tumor is a locally destructive mass of predominantly osteoclast-like, multinucleated giant cells in a vascular and fibrous stroma consisting of spindle-shaped fibroblasts and hemosiderin-filled macrophages. The focal areas of bone resorption ultimately coalesce to visible cysts. Microscopically, cortical and trabecular bone is replaced by loose connective tissue. The tumors are soft and brownish in color due to episodes of hemorrhages from microfractures of the thinned and demineralized bone. They present as either single or multiple lesions and are destructive by their lytic and expansive properties [5, 14, 15]. 

While SHPT is common, a diagnosis of brown tumor is unusual, with an estimated incidence between 1 and 2%. However, the diagnosis is easily overlooked. A radiologic lucency without associated pain, local compression, or fracture is dismissed as a simple bone cyst. More importantly, the formation of these tumors requires unremitting osteoclastic activity without associated bone formation, i.e., without the appropriate osteoblastic differentiation. The skeletal response to PTH can be either catabolic or anabolic depending on a diverse array of inﬂammatory cytokines and chemokines (RANKL, IL-6, TNF-α, and others). Pulsatile (physiologic) release of PTH stimulates osteoblasts through binding at the PTH1 receptor (PTH1R) [16]. Osteoclasts do not express PTH1R; rather, stimulation of osteoclasts occurs via paracrine effects from osteoblasts [17]. Therefore, since brown tumors consist mainly of collections of osteoclasts within a fibro-vascular bed, the formation of such a brown tumor requires a cluster of autonomous, self-sufficient osteoclastic cell lines that no longer rely on the paracrine effects of osteoblasts [18]. 

Brown tumors can be localized in any skeletal bone; however, most published reports are in dental and oral maxillofacial journals due to a high prevalence for the mandible, maxilla, and hard palate [14, 15]. They are also seen in the ribs, pelvis, and femur, but spinal lesions are infrequent [19, 20, 21]. The clinical manifestation of a brown tumor varies depending on its location. Most are asymptomatic, while others can cause local swelling, protuberance, or even disfiguration, especially in the face. Pain is due to the tumor per se, or can be a sequela of the disease process, for example, pain due to a pathologic fracture of the hip or pelvis. If the location is in the vertebral column, radicular pain or paresthesia may occur. More severe manifestations such as cauda equina syndrome, paraparesis, and even paraplegia constitute an acute neurological emergency [5, 22, 23, 24]. 

The first report of a spinal brown tumor was published in 1968, which was due to primary HPT [25]. We found only 16 published cases of dialysis patients with symptomatic brown tumors involving the spine published between 1977 and 2019. In those case reports, there was no significant difference in gender, age, or duration of dialysis, which varied from 1 to more than 10 years. 

Brown tumors do not have specific laboratory or imaging findings. Most commonly, patients have marked elevations of PTH, phosphorus, and often calcium. On imaging, the lesions can mimic a host of other tumors such as myeloma, giant cell granulomas, or metastatic lesions, in particular from breast or prostate cancer. Simple bone cysts and depositions of amyloid (β-2 microglobulin) in ESKD can also mimic brown tumors [5]. In the vertebral column, CT imaging reveals osteolytic and expansive soft tissue masses that replace the cancellous bone. MRI can better delineate the degree of neural compression, if present. Brown tumors are typically hypointense on T1-weighted imaging, and hyperintense on T2 [19, 20]. 

The appropriate management of brown tumors is often difficult, and there are no societal recommendations to help guide management. There is no mention of brown tumors in published guidelines (Kidney Disease: Improving Global Outcomes (KDIGO) 2009 and 2017 Update, Kidney Disease Outcomes Quality Initiative (KDOQI) 2003) [3, 26, 27]. There is also no discussion of brown tumors in standard nephrology textbooks, at best a cursory note. 

Brown tumors in advanced CKD and ESKD are almost invariably associated with severe SHPT. The focus in management involves an aggressive lowering of PTH by treating hyperphosphatemia, hypocalcemia, and vitamin D deficiency. This involves a reduced dietary phosphate intake, meticulous use of phosphate binders, the addition of native and active vitamin D, and most importantly, calcimimetics. 

The increasingly prominent role of calcimimetics was emphasized in the KDIGO Update 2017. While recognizing the negative results of the EVOLVE trial – a non-significant reduction of the unadjusted primary composite endpoint (death and non-fatal cardiovascular events) –, they nevertheless emphasized cinacalcet’s importance in the treatment of severe SHPT [28]. Indeed, a post hoc analyses highlighted cinacalcet significantly reduced progression of SHPT to “severe unremitting” HPT (defined as two consecutive PTH values over 1,000 pg/mL, hypercalcemia, the use of cinacalcet with hypercalcemia, or patients requiring PTX) [29]. While it is recognized that cinacalcet may produce significant hypocalcemia, some degree of asymptomatic hypocalcemia is permissible in this population. Thus, cinacalcet is a cornerstone in managing SHPT. 

PTX appears to be the best option after medical management fails, though the indication for PTX remains somewhat vague. The KDIGO 2017 Update did not change the KDIGO 2009 statement (a grade 2B recommendation indicating, “we suggest” and a “moderate” quality of evidence) that “if PTH levels remain markedly elevated or continue to rise, the patient has failed medical/pharmacological therapy, we suggest PTX.” Similarly, the KDIGO guidelines do not define what constitutes “severe” in SHPT. In EVOLVE, “severe” HPT was arbitrarily defined as > 1,000 pg/mL (median PTH level of 1,872 pg/mL). To put this into perspective, KDIGO recommendations for target PTH level in persons with ESKD have remained unchanged since 2009, aiming to “maintain iPTH levels in the range of ~ 2 – 9 times the upper normal limit for the assay.” The level of recommendation is 2C (“we suggest” and a “low” quality of evidence). Since a typical range of a “normal” PTH level is 15 – 80 pg/mL, the upper limit of a goal PTH level is up to 720 pg/mL. In reality, however, many clinicians and experts in the field will not commit to a particular lab value, as studies have shown no rationale for a special goal [Geoffrey A. Block, personal communication]. Rather, “progressively rising” or “persistently high” PTH levels warrant consideration for PTX. There are data and reports demonstrating the benefits of PTX with re-mineralization of bone and regression of the tumors just months following the surgery [30, 31]. 

In our case, we decided on prompt neurosurgical intervention as PTX alone would not relieve the cord compression at T3 with the risk of paralysis, nor alleviate her pain. She underwent urgent surgical resection of the tumor at T3, followed by PTX. No intervention was performed at the T12 lesion, as this was not deemed urgent. 

**Figure 5.**
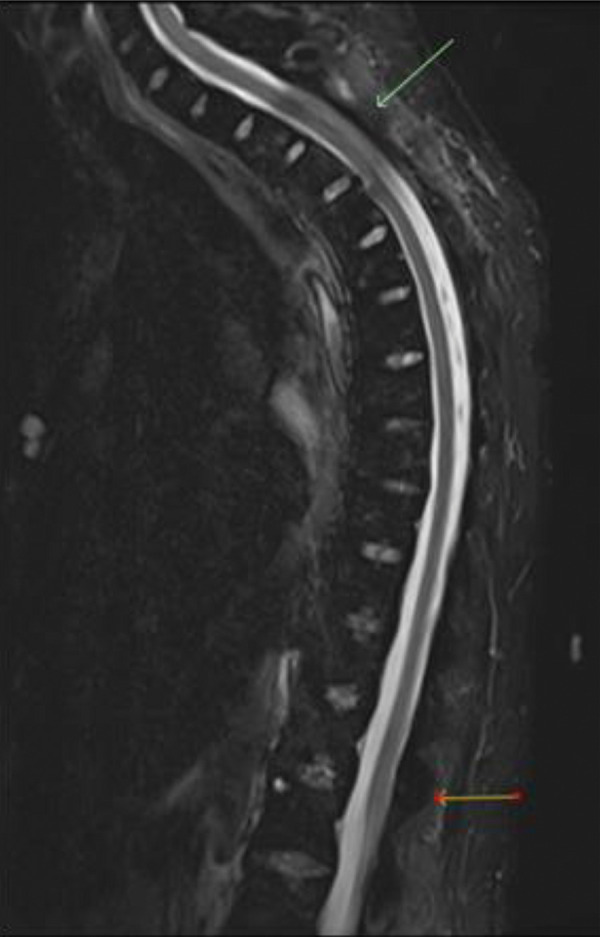
Repeat MRI 3 months after resection of the T3 lesion and PTX shows stable resection site at T3 and regression of T12 tumor.

**Figure 6.**
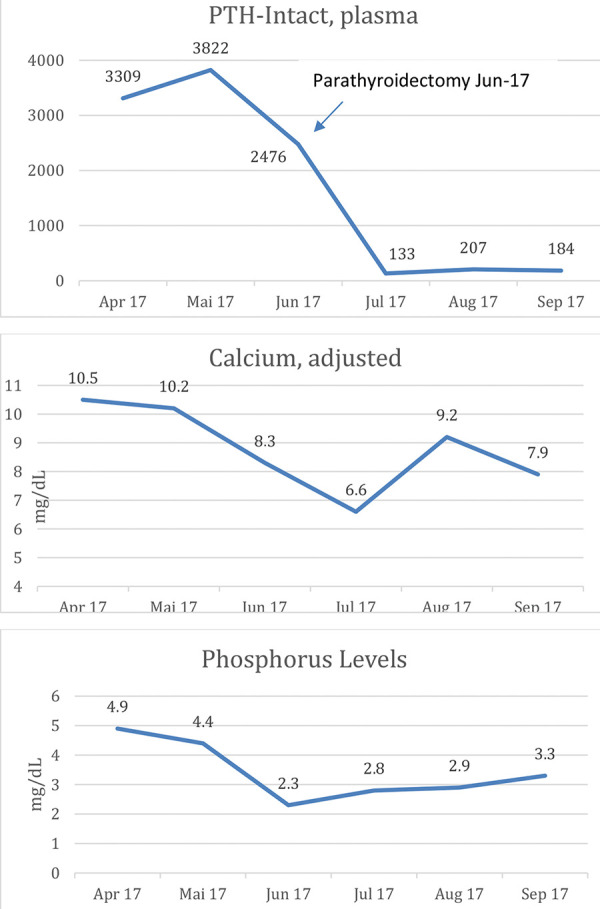
PTH, calcium, and phosphorus levels before and after PTX.

A few months after spinal decompression and PTX, repeat MRI showed no recurrence at T3, and as expected, regression of tumor at T12 (Figure 5). In terms of her laboratory parameters, PTH is currently at goal in the 200 range; both calcium and phosphorus are at target with a combination of calcium-containing phosphate binders, calcium supplementation, vitamin D, and calcitriol (Figure 6). 

Our case illustrates a rather dramatic presentation of a brown tumor. Aggressive management led to a successful outcome. We believe brown tumors are both under-recognized and under-reported, and hope for discussion and guidance by expert working groups in the future. 

## Funding 

None. 

## Conflict of interest 

None.
